# Renewable resilience in conflict: lessons learned from Syria’s solar-powered electric health vehicles

**DOI:** 10.3389/fpubh.2025.1560063

**Published:** 2025-04-02

**Authors:** Ahmad A. Alnasser, Mohammad Darwish, Ahmed Aldbis, Camila Polinori, Shatha Elnakib, Saverio Bellizzi, Daher Zedan, Bara Zuhaili

**Affiliations:** ^1^Johns Hopkins School of Medicine, Baltimore, MD, United States; ^2^Center for Humanitarian Health, Johns Hopkins Bloomberg School of Public Health, Baltimore, MD, United States; ^3^Union for Medical and Relief Organizations (UOSSM), Gaziantep, Türkiye; ^4^Department of Medical, Surgical and Experimental Sciences, University of Sassari, Sassari, Italy

**Keywords:** solar power, humanitarian access, renewable energy, conflict zone, Syria

## Abstract

The Syrian Civil War has resulted in significant devastation, including widespread displacement of millions and extensive damage to infrastructure, particularly healthcare infrastructure. Attacks on healthcare facilities have been frequent, leading to a drastic reduction in available medical services and the exodus of healthcare workers. The resulting impact on civilians, compounded by energy shortages, has been severe, limiting access to essential services. In response to these challenges, the Union of Syrian Medical Relief Organizations (UOSSM) has introduced the solar-powered electric vehicles for healthcare transportation in Northwest Syria. This development is a significant step toward sustainable energy solutions in conflict zones, providing a reliable source of power for essential services such as healthcare delivery. In this paper, we document this experience which underscores the importance of local involvement and partnerships in implementing such solutions, as well as the need for broader research and education initiatives to ensure the long-term viability of renewable energy systems. These initiatives allow for a sustainable future solution while enabling local actors to participate in their development and maintenance. By prioritizing sustainability and autonomy, initiatives like UOSSM’s solar-powered electric car demonstrate the potential for innovative responses to complex humanitarian crises around the world, following Syria’s example.

## Introduction

The Syrian conflict, which began in 2011 during the Arab Spring, has resulted in one of the most devastating humanitarian crises of the 21st century. Over 6 million people are internally displaced, while 5.5 million have fled the country as refugees (1, 2). The civil war has decimated over half of Syria’s health facilities, many targeted deliberately, forcing healthcare workers to flee amid documented kidnappings, torture, and executions (3, 4). Field hospitals, often established to replace destroyed facilities, are frequently attacked and must relocate constantly (5). Combatants have looted ambulances and used siege tactics to block medical aid, worsening the crisis (6). Vulnerable populations, individuals with disabilities, face significant barriers to accessing health care locally or abroad (7, 8). The sustained attacks on health have eroded the health system, leaving millions without essential services and a substantial decline in energy infrastructure (9).

Only one-third of the global population has access to electricity, and blackouts are common due to capacity shortages and unreliable infrastructure stemming from high costs (10). This disparity is heightened in conflict zones, making these areas particularly vulnerable to energy insecurity. In Yemen, an already-existing energy shortage was exacerbated by the current war, which led to the demolition of more than half of the electrical power infrastructure (11). In Afghanistan, the total power supply consisted of isolated grids powered largely by imported diesel and furnace oil, a costly and inconsistent source (12). Especially as conflict increases further ravage these systems, renewable energy solutions become more necessary. Energy insecurity in conflict zones directly impacts public health systems by disrupting essential services such as vaccine cold chains, emergency transportation, and health facility operations. The lack of reliable energy sources contributes to delays in care, higher mortality rates, and diminished capacity for disease prevention and treatment (13).

Renewable energy solutions, such as solar power, offer a sustainable approach to bridging these gaps, ensuring continuity of care in fragile settings (14). Traditional diesel-powered ambulances often face challenges such as fuel scarcity and high operational costs, exacerbated by disrupted supply chains (15). Hybrid models, while reducing fuel dependency, require complex infrastructure that may be unfeasible in unstable regions. Electric vehicles dependent on grid power are similarly hindered by unreliable electricity supplies (16). In contrast, solar-powered vehicles offer a decentralized and sustainable solution. They capitalize on abundant solar resources to ensure continuous operation without the vulnerabilities associated with fuel logistics or grid dependencies (17, 18). Case studies from Yemen and Gaza have demonstrated how solar microgrids provide uninterrupted power to health facilities, enhancing service reliability during crises (19–22). By decentralizing energy systems, solar technology reduces dependency on vulnerable infrastructure and mitigates the health consequences of energy shortages (13). Likewise, studies have demonstrated that integrating solar energy into humanitarian settings can lead to significant cost savings and environmental benefits. For instance, a case study on solar-diesel hybrid mini-grids in Nyabiheke refugee camp, Rwanda, found that these systems could reduce total energy costs by up to 32% and decrease emissions by up to 83%, with cost payback times ranging from 0.9 to 6.2 years (23).

In November of 2021, Union of Syrian Medical Relief Organizations (UOSSM) launched the first electric vehicle powered by solar energy in Northwest Syria, following an ongoing effort to strengthen solar energy infrastructure in areas with limited energy access. In this paper, we first present the effects of conflict on energy infrastructure, with a focus on consequences on the health system and civilian life. Secondly, we describe UOSSM’s experience with implementing an innovative intervention to advance sustainable energy solutions in Syria and discuss its implications for healthcare delivery. Despite the growing interest in renewable energy solutions within humanitarian contexts, there remains a significant research gap concerning their direct integration into health systems operating in active conflict zones. Existing studies have primarily focused on energy access in displaced populations and the role of sustainable energy in humanitarian actions (24). This paper aims to bridge this gap by examining the implementation of solar-powered transportation within a war-affected region’s health care infrastructure, providing empirical evidence on its feasibility and impact. By highlighting the advancements in bringing electricity to energy-poor areas, we draw out lessons in how we can leverage sustainable energy solutions to more efficiently respond to humanitarian disasters.

### Existing literature and research needs

There is a notable gap in the field focusing on renewable energy use in disaster and conflict areas, and their potential applications to sustainability. Existing literature has established several important foundations in related areas. Ratterman and Garwood (25) conducted a study on the use of solar energy to power remote clinics in Burma, highlighting the utility of renewable energy in resource deprived areas. Multiple studies, similarly, have investigated solar power’s role in vaccine storage, proposing and implementing collaborative models with local hospitals to strengthen distribution networks (26, 27). The deployment of solar energy containers in eastern Chad has dramatically reduced reliance on traditional generators, ensuring uninterrupted medical services for over 50,000 refugees (28). Similarly, the integration of solar-powered clinics in Uganda has expanded medical care access in off-grid refugee settlements (29).

Recent initiatives have also highlighted solar energy in terms of health transport services in humanitarian settings. For instance, Siddique et al. (30) proposed the usage of a solar-powered rickshaw ambulance based on experiences in Bangladesh, emphasizing a pressing need to access remote populations in a sustainable and cost-effective model. While the proposal was sound and identified an important need, a follow-up study on its implementation has yet to be published.

However, while these studies illustrate the viability of renewable energy in a healthcare setting conceptually, as well as the importance of solar energy to access, there are currently no published studies examining the implementation of solar-powered transport in active conflict settings (31). Given the urgent need for reliable and sustainable healthcare delivery in such settings, addressing this gap in the literature is essential.

### Impact of conflict on energy infrastructure and health systems

Before the war in 2011, Syria was one of the largest energy producers in the Middle East, producing around 380 thousand barrels of oil a day (13). While conflict-related destruction has significantly impaired Syria’s energy infrastructure, sanctions have further constrained the ability to import essential components for energy system repairs (14). Access to electricity has thus consistently been ranked among the top priority needs reported by Syrians in the United Nations Multi-Sectoral Needs Assessment (32). In early 2016, as government forces began to regain control of the oil and gas fields, it improved the production of natural gas, although nowhere near pre-conflict numbers. Similarly, all three of Syria’s major hydroelectric dams were damaged in the conflict in the electrical and control elements, rendering the power supply originally distributed across the country at about 40% of its total capacity (33–36). This impacts daily life, where in 2021 individual consumption of electricity was 15% of what it was in 2011 (33). 5.3 million people are living with less than 2 h of public electricity per day.

The energy consequences of conflict in Syria mirror other energy challenges in other conflict-affected settings. In South Sudan, protracted civil wars spanning decades have led to severe energy service depletion, particularly in access to water (37, 38). As a result, only half of the population has been able to access safe water reliably, having to rely on open wells and unsafe water sources like rivers and ponds. Similarly, with the escalation of violence in Yemen since March 2015, water and electricity infrastructure has deteriorated severely with major damage to ports and blockades (39). The trade blockades caused price hikes for tankers and diesel, which also led to electricity blackouts that affected the delivery of water (38). In 1989, the Farabundo-Marti National Liberation Front was able to sever service provision to nearly 90% of El Salvador through attacks on 10 power plants, even writing manuals on how to attack power supply (40). Overall, attacks on energy systems represent a worldwide issue, and the downstream impacts on civilian life necessitate a sustainable solution.

Health systems may also be direct targets in conflict. In Syria, the aerial bombardment of health facilities, in addition to damaging physical infrastructure, has disrupted the referral system and the ability to provide care to patients in remote areas. In addition to contravening international law, these attacks function to stop access to healthcare, forcing civilian displacement (4, 41). This can be seen in eastern Aleppo, which in late 2016 reported that all functioning hospitals had been attacked, and minimal healthcare workers remained in the area. Compared to the nearly 30,000 doctors in Syria in 2009, it has now been depleted to around 15,000 as of 2024, and up to 70% of healthcare workers have fled the country (1, 42). Of the healthcare providers who remained, the prevalence of psychological distress is around 52%, and levels of burnout among residents were found to be 93.75 in 2019 (43).

### UOSSM’s innovation: the solar energy-powered electric car

**Figure 1:**
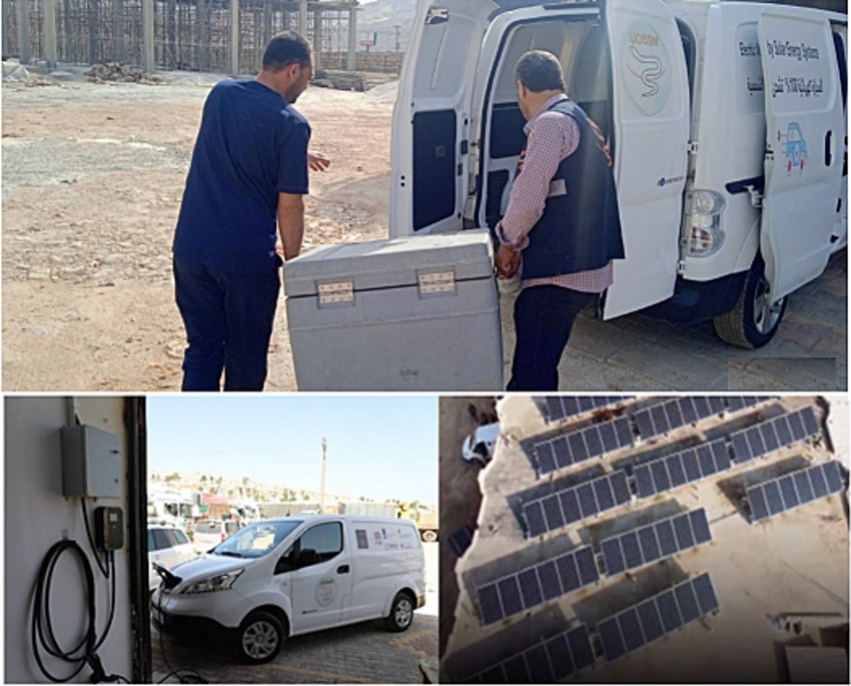
Images of the solar-powered electric vehicles implemented by UOSSM, charging stations by within the hospital area, and solar panels.

On November 30, 2021, Union of Syrian Medical Relief Organizations (UOSSM) launched the first electric vehicle in Northern Syria, for usage in vaccine transport as well as patient transport through the referral system (44). This vehicle, utilizing a lithium-ion battery, can be charged at health facilities, which have previously been converted to utilizing solar energy by UOSSM’s Syria Solar Initiative (SSI), part of the broader Health Integrated Resilience System (HIRS) program. In its entirety, this development means that transport can be recharged without the dependency on an unstable fuel and electricity supply (Figure 1). Furthermore, it allows humanitarian actors to deliver critical care more frequently in a conflict zone, saving scarce fuel supplies for under-resourced hospitals. UOSSM was founded in 2012 in Geneva and has been working on expanding solar energy capabilities for hospitals throughout Syria since 2017.

The development of an electric vehicle was part of a three-pronged approach within the HIRS program, alongside increasing solar energy access to hospitals and facilitating Humanitarian Telemedicine programs to allow more people in Syria to access healthcare. The official project period launched on April 1, 2019, and the main delay was due to sanctions, which prevented cars of this nature from entering Syria. Compounded to supply, the sanctions resulted in increased costs compared to the global average price of cars. Overall, the electric vehicle cost USD40,000, and its modifications, such as rear seat modifications for patient transport, amounted to an additional USD5000, compared to a fuel-powered vehicle cost of USD30,000 (Table 1). The car itself consumed 1,278 kWh in total, around 0.17 kWh per km, marking a notable cost reduction compared to a standard fuel car (0.22 kWh/km).

**Table 1:** Comparison of conventional fuel-powered vehicles and solar-powered electric vehicles for ambulance usage in Northwest Syria.

Metrics	Conventional fuel-powered vehicles in NW Syria	Solar-powered electric vehicles in NW Syria
Initial Vehicle Cost	$30,000	$40,000
MODIFICATIon costs for medical equipment	N/A	$5000
Cost per distance ($/KM)	$0.13	$0.05
Operation and maintenance Costs (monthly)	$171.10	$83.70

NW, Northwest; costs in USD.

Data collection for this study involved systematic tracking of vehicle operations over a 30-month period from the official implementation in November 2021 to May 2024, with a focus on metrics such as the number of patient transports, distance covered, and energy consumption. Patient transport logs were maintained by UOSSM. These logs have recorded each trip’s purpose, duration, and outcomes. Energy consumption data was gathered via a battery management system that captured real-time solar energy generation and usage statistics. Cost-effectiveness was evaluated by comparing operational expenses of solar-powered vehicles to traditional fuel-powered alternatives considering factors like maintenance costs and fuel savings.

For the duration of the project the total distance crossed by the vehicle was 7,442 Km for 476 trips, averaging about 15.6 Km per trip. In total, the number of transported vaccine beneficiaries was about 132,000, while 43 total patients were transported. In the 6 months before the end of the study period, the monthly average of beneficiaries was about 22,000. Of the 476 trips, 303 were below 200 kg, 140 were between 200 and 400 kg, and 8 were more than 400 kg, including the vaccines, patients, and staff. This marks a significant and energy-efficient mode of transportation, bringing an important amount of cargo for better patient care. In addition, because the car did not need fuel to operate, this allowed for the reallocation of fuel resources to service other parts of essential operations at the hospitals.

**Figure 2:**
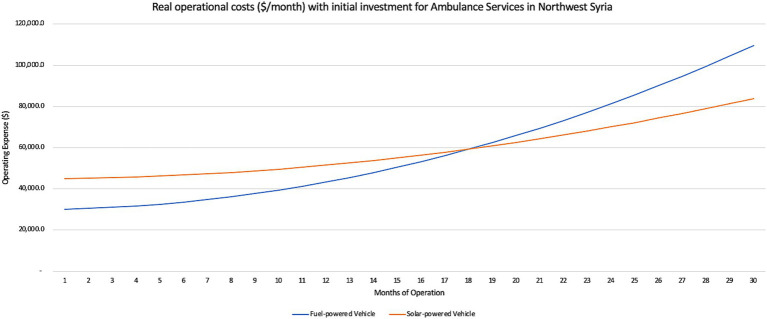
Monthly operational costs with initial investment (USD) of solar-powered electric ambulance services in Northwest Syria.

**Figure 3:**
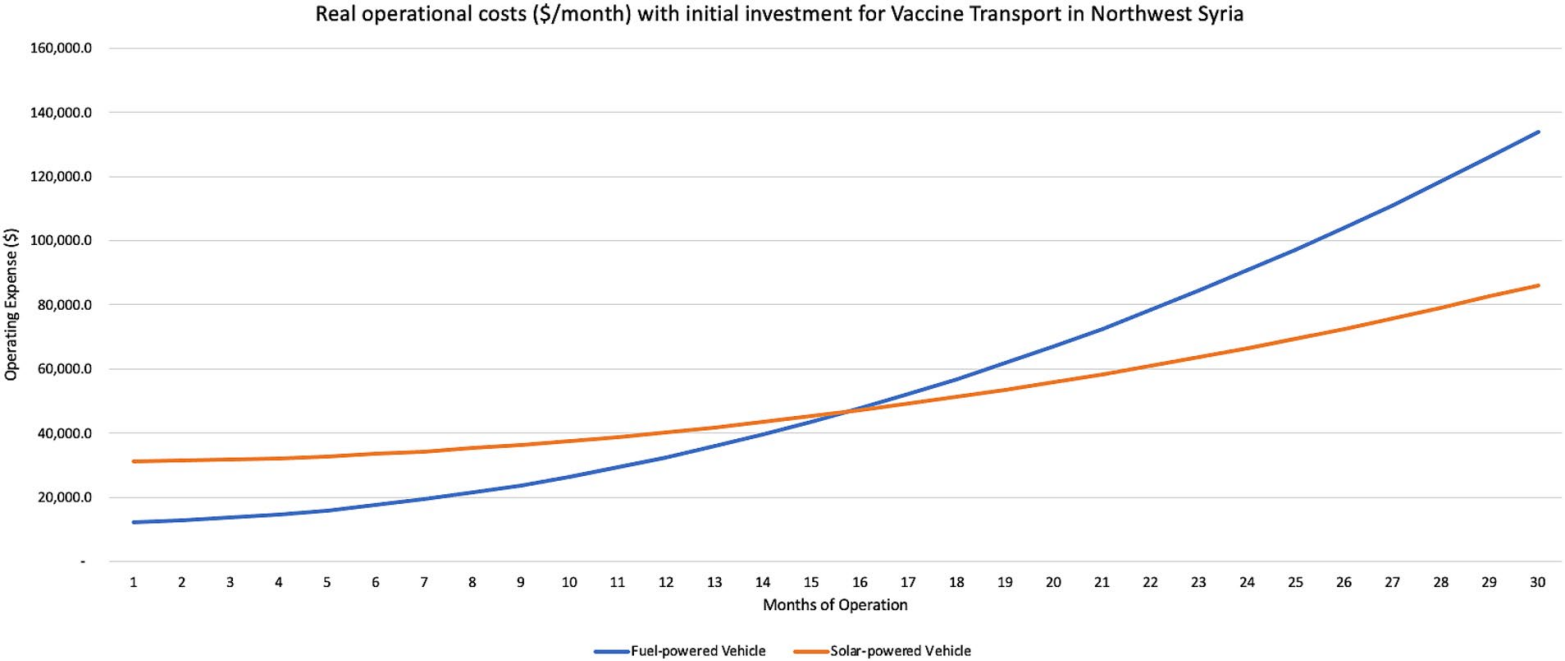
Monthly operational costs with initial investment (USD) of solar-powered electric vaccine transport services in Northwest Syria.

The solar-powered electrical vehicle also managed to have fewer cumulative operating expenses than fuel costs within 18 months, for both ambulance and vaccine transport services (Figures 2, 3). A similar distance with a fuel car for ambulance services averaged about USD1091.00 in total, or USD0.13/km while the electric vehicle charged on the solar PV network only cost USD259.00 in total, or USD0.05/km. For vaccine transport, the same distance in a fuel-powered vehicle cost USD0.12/km while the electric vehicle charged on the solar PV network only cost USD0.05/km.

### The benefits of renewable energy development in conflict areas

Successful renewable energy adoption in conflict settings requires local engagement at all levels. For instance, in North-West Syria, local engineers and technicians have been trained to install and maintain solar systems, reducing dependence on external actors and increasing system resilience (45, 46).

The solar-powered electric vehicle represents the capacity for advancements in sustainable technology, both in Syria and internationally, especially where infrastructure may be at risk or compromised due to ongoing conflict. Syria is a suitable candidate for such a pilot program, given its geography and sun exposure for long hours. The country has the second-highest solar radiation in the Middle East (33, 47, 48). However, despite the effectiveness of this renewable energy source, there are not many projects currently underway to expand Syria’s solar capabilities. Thus, in addition to a pilot program, UOSSM’s SSI represents an investment in integrative solutions in conflict settings that promote environmental sustainability and mitigate the dependency on traditional fossil fuels.

Investing in solar energy, from a cost perspective, is increasingly favorable compared to traditional fuel sources. Projections have the cost of electricity per megawatt generated from renewables as now cheaper than imported fuel (49, 50). Similarly, a UNDP report of a recent project to increase solar power access in Yemen found that it reduced energy costs by 65 percent, and increased the rate of accessing uninterrupted electricity to nearly 75 percent (11, 51). This is especially true in countries like Syria, which have ample solar resources, which bring an already cost-effective10–12 US¢/kWh cost of solar energy in America and Europe down to approximately 6 US¢/kWh (52). For reference, diesel generators in some countries can extend to 50 US¢/kWh or more, while the most efficient carbon-emitting forms of electricity production can be as low as 8 US¢/kWh (53, 54). Despite absolute funding for humanitarian relief constantly increasing, the increase is not enough to keep up with Humanitarian Response Plans (HRPs) in increasingly more complex and larger-scale disasters (55). The COVID-19 pandemic also exacerbated costs while limiting the capacity for physical movement within conflict, stretching the divide between funding and costs even further (42). Thus, having a sustainable solution that also trims costs in the long term is necessary for any successful intervention. The transition to solar energy circumvents the exploitative pricing of private generators, while also bypassing the supply issues that accompany developments in fighting and sanctions, in total saving around 1.8 billion dollars annually (41). In Morocco, the transition to renewable energy by 2040 was accompanied by a simulated employment potential of 269,252–499,000 new jobs (56). In addition, the long-term cost savings for the system also for the expansion of PV solar systems to remote areas, as was the case in Nepal, effectively paying for themselves (31, 57). Typically, investors are reluctant to donate to unstable regions, given the unpredictable nature of conflict, as was the case in Libya and Yemen (58). However, in the OPT, a similar partnership between the Palestinian Energy and Natural Resources Authority, PENRA, and international organizations created solar energy grids for hospitals in Gaza as an alternative to importing fuel for generators. The cost, typically a range of $6–USD 10 million, decreased to USD 4 million for installing 34 critical units within 10 hospitals in 2018 (59, 60). These international areas, like Syria, stand to benefit strongly from a push toward renewable energy, and already have the basis for an initiative like UOSSM’s.

Similarly, the environmental impacts of more renewable energy sources mean that increased energy demands can be met, in line with international agreements on limiting climate change. The Paris Agreement set a goal for limiting the global mean temperature increase to 1.5 degrees Celsius. A switch to renewable energy, combined with the increase in energy efficiency, can account for 90% of the required CO2 reduction by 2050 (58). Empirical studies conducted on the effect of renewable energy consumption on logistical performance concluded that renewable energy significantly enhances logistical operations with better environmental sustainabilities (61, 62). Public health expenditure is also a reduction downstream from adopting renewable energy practices since using polluting energy in vehicles and businesses causes poorer air quality, which can exacerbate the incidence of diseases like asthma, and brain, and lung cancer (63). This, in turn, reduces productivity and labor efficiency. Therefore, these environmental impacts make solar projects especially attractive to international donors and development agencies seeking to invest in sustainable, climate-resilient infrastructure. Donor investment in solar energy not only addresses immediate energy needs but also supports long-term environmental and economic sustainability, highlighting the dual benefits of reducing emissions and lowering operational costs and strengthening the case for funding (64). The use of renewable energy in environmental practices has attracted foreign investments, giving more significant export opportunities in addition to a more positive image of the country (61).

Utilizing solar-powered healthcare transport can extend these developments into other sectors, allowing for a range of benefits even beyond healthcare. Solar-powered healthcare transport and infrastructure not only provide clean, reliable energy for medical services but also catalyze improvements in emergency response, education, and water purification. While energy itself is not in the humanitarian cluster system, its provision extends beyond individual sectors into a nexus of interconnected benefits, especially in conflict settings where they may be disrupted (24). As shown in UOSSM’s implementation, using solar energy for vehicle transport, a strong cross-sector benefit is the enhancement of emergency response capabilities. In conflict zones, where traditional energy infrastructure is often unreliable or destroyed, solar-powered transport and communication systems are vital. Solar-powered ambulances or mobile health units ensure continuous operation, even when fuel supplies are scarce or disrupted. These vehicles can transport patients, medical supplies, and personnel efficiently without relying on diesel. Additionally, solar-powered communication systems provide reliable, real-time coordination between first responders, healthcare facilities, and crisis management teams, improving response times and potentially saving lives (65, 66). Solar-powered systems can also significantly enhance the efficiency and sustainability of water and sanitation services by providing cost-effective, reliable power for water pumping, purification, and hygiene facilities (67, 68). Solar-powered water pumps and purification systems ensure a steady supply of clean water, reducing the prevalence of waterborne diseases and supporting hygiene practices. They reduce dependency on costly and logistically challenging diesel fuel, with studies demonstrating substantial long-term cost savings and rapid payback periods in refugee camp settings (24, 69). Furthermore, solar-powered lighting at WASH facilities improves safety, particularly for women and children, by reducing the risk of violence during nighttime use (70). Renewable energy can significantly benefit the education sector in humanitarian settings by providing reliable power for schools and learning environments. Access to electricity enables extended study hours, supports the use of modern learning technologies such as computers and projectors, and helps schools remain operational beyond daylight hours (53).

Reliable energy also reduces the financial burden of fuel and can help attract and retain teaching staff, improving education quality and accessibility (71, 72). Schools can thus serve as hubs for adult education, vocational training, and community meetings, fostering long-term recovery and social cohesion given the extension of services. By addressing critical needs in emergency response, education, and water access, solar power fosters resilience and sustainability in conflict settings. These cross-sector benefits not only improve immediate living conditions but also lay the groundwork for long-term recovery and development. Thus, solar energy represents a strategic investment for donors and policymakers seeking to maximize their impact in fragile and conflict-affected regions (Table 2).

**Table 2:** Comparison of conventional fuel-powered vehicles and solar-powered electric vehicles for vaccine transport usage in Northwest Syria.

Metrics	Conventional fuel-powered vehicles in NW Syria	Solar-powered electric vehicles in NW Syria
Initial vehicle cost and modification	$12,000	$31,000
Cost per distance traveled ($/km)	$0.12	$0.05
Operation and maintenance Costs (Monthly)	$262.20	$118.50

NW, Northwest; costs in USD.

### The future of energy: scaling and expanding

To scale such initiatives, stable supply chains for solar panels, batteries, and inverters must be ensured. Lessons from decentralized solar microgrids in fragile states, such as those implemented in refugee camps in Jordan, highlight the potential for regional energy networks to improve sustainability and cost-efficiency (73, 74).

In the future of the renewable energy landscape, it is imperative to utilize community involvement and combine it with sustainability, allowing for cost-cutting measures that concurrently ensure that hospitals remain powered. It is evident that the conflict in Syria led to lower stores of natural gas and petroleum fuels, but a larger demand for electrical energy than available. Thus, the need for diverse, renewable energy is important, and that can set the stage for future conflict zones (75). Solar energy systems can be decentralized and interconnected, meaning that individual households (or hospitals) can have solar panels as singular entities, and an attack on a central power-generating plant will not lead to widespread outages (38). In addition to allowing more consistent electricity usage, distributed solar energy systems offer a decentralized approach to electricity generation, particularly in remote and off-grid communities. By installing rooftop solar panels on residential and commercial buildings, as well as standalone solar microgrids in rural areas, Syria can expand access to electricity, improve energy resilience, and empower local communities to become active participants in the energy transition (76).In qualitative studies, individual respondents reported satisfaction with the system meeting their basic needs, but both individuals and hospital systems reported inconsistency in electricity supply as well as not being fully aware how to operate and maintain their existing systems (77, 78).

This represents a point of interest in future developments, mainly in education of these systems and future jobs for solar engineers and repair workers. Autonomy and positive feels over energy decisions are important for the success of solar energy throughout Syria as well as for perceived success among the people (79). UOSSM is currently among the international NGOs committed to providing this, with solar-dependent hospitals being launched in 2017 and 2019 accompanied by training programs to increase capacity and allow for maintenance of the solar systems (80). Typically, investment costs in renewable energy are higher in developing countries due to poorly trained labor forces, and a need to bring in materials or engineers from abroad to aid in maintenance of renewable generation systems. However, in developing countries, solar energy has been considered a significant job-creator and particularly stable given building, operational, and maintenance phases (18). For example, Practical Action’s RE4R, can be used as a model for future vocational training in a humanitarian setting. Following its three-year duration, Syrian refugee settlements in Jordan received solar water heating, expanded solar PV systems for 14 schools containing 12,400 children, and training opportunities to over 100 youth in a renewable energy vocational course (81). A vocational course, like that one, could bolster sustainability through building local expertise. Providing practical training, such as PV pump installation, maintenance of systems, and battery management. Even more, establishing local expertise allows for future iterations of training to be done locally as well, as was seen in a women’s Solar PV vocational training program in Uganda (82). The Syrian example provides an example of the capacity of these programs to aid in actionable goals toward sustainable energy, and practical training allows for humanitarian interventions to further develop.

Attention should also be directed toward long-term sustainability, including ways to lower costs of manufacturing and development of these vehicles. In Syria, UOSSM imported necessary materials, like the battery and appliances for the electric car, and retrofitted them for use with solar power sources. In addition to the delivery of the car itself, these materials may be subject to supply chain and potential maintenance issues as a result of sanctions. Humanitarian Aid in Syria is currently dominated by international humanitarian organizations, and historically only around 1.2% of international aid is directly allocated to local NGOs (83). The push toward localization more broadly has been driven by Syrian NGOs, in partnership with local health directorates, but has been met with reluctance from risk-averse donors and reluctance of international agencies to cede control (84). Yet, localization is a necessary step for the further development of initiatives like the solar-powered electric vehicles, as it saves on significant overhead costs, while utilizing the ingenuity and deep knowledge of power structures that local actors possess. Thus, the expansion of solar powered electric vehicles necessarily requires organizations like UOSSM to fill in the gaps that international humanitarian agencies are overseeing, for they can train local actors and build long-term connections within Syria. As can be gleamed from UOSSM’s solar powered electric vehicle implementation, there is a startup investment that needs to be made initially with such a project (the initial price of the vehicle combined with modifications for patient and vaccine transport), but it can yield greater savings than its fuel counterpart just 18 months following implementation.

Another option to scale this development in Syria while being conscious of the humanitarian landscape is to utilize humanitarian exceptions in order to receive supplies. Humanitarian exceptions refer to any special legal or regulatory allowances that enable the delivery of essential goods and services to a population in need (85). This applies in the context of Syria given the sanctions imposed on the country, as well as trade restrictions between numerous UN member states and Syria. These exceptions can allow for humanitarian aid to reach a vulnerable communities, including energy and tools for energy infrastructure (86). These exceptions, in addition to the inclusion of local actors in the development and organization of interventions, can create a sustainable network of manufacturing and development of solar powered electric vehicles in Syria, independent of international donors and self-sustaining. In practice, this would mirror the current arrangement of Syrian aid in Northwest Syria but expanded further to include local actors. This means that on the ground, actors like UOSSM, Syrian American Medical Society (SAMS), and Syria Relief and Development (SRD), would facilitate the training and supply for local partners (87), and leverage the humanitarian exceptions to build self-sustaining systems.

Expanding on this development for future networks of solar-powered vehicles and decentralized solar grids creates a resilient post-conflict energy network to build from. The benefits of a decentralized grid are twofold: (1) there is less reliance on a single or a few, large generators which can be significant targets during armed conflict (88), and (2) the inclusion of otherwise remote or rural populations given their installation. Therefore, in the short term, stakeholders could roll out multiple solar-powered electric vehicles, which hospitals can use for battery charging to facilitate patient and supply transport through conflict-ridden areas. In parallel, authorities could implement a phased rollout of decentralized solar grids. Initially, teams could install micro-grids in key locations such as hospitals, schools, and refugee camps to provide reliable electricity for essential services. As stability improves, these grids can expand to connect households and small businesses, fostering local economic development and improving quality of life. Eventually, policymakers could link these micro-grids to form a national decentralized energy network, which would enhance resilience and reduce the risk of widespread power outages. In a related example, a World Bank GIS analysis found that it is possible to provide electricity to 55–73% of Afghanistan’s population by adding off-grid systems (12, 89). This strategy not only addresses immediate energy needs but also lays the groundwork for a sustainable energy infrastructure that aligns with global climate goals. Given the capability of countries with similar energy landscapes as Syria to utilize alternate forms of energy, it stands to reason that these lessons learned from Syria on implementation could generate future innovations in these nations.

Likewise, to ensure scaling and improvement over time, the strategic use of data will be crucial. Robust data collection on energy usage, vehicle efficiency, and healthcare outcomes can provide valuable insights to refine operations and improve resource allocation. For instance, monitoring vehicle performance and energy consumption can help identify areas for technological improvements or maintenance needs, ensuring the long-term viability of the project. Due to security constraints within conflict zones, the collection of reliable data can be hindered or stopped altogether, which is why it is important to incorporate local structures into the data collection process. Through participation of communities, data can be collected on the ground and reciprocally findings can be used to elicit change and improvements for a given intervention (38, 90). Community involvement can bring infrastructure sustainability, while also building trust for long-term solutions. This is especially the case, given that data is not routinely collected on energy, and there is no standardization for measuring and reporting energy data (91). To enhance data utilization, partnerships with local universities and research institutions should be explored. In Syria, academic research lacks infrastructure for a multitude of reasons, including lack of structural and financial support, access restrictions to collaborative spaces, and limited application of their findings in a rapidly changing environment (92, 93). Collaborative research on renewable energy applications in conflict zones can yield innovative solutions and provide a robust evidence base for scaling efforts. Data-sharing initiatives can also facilitate knowledge exchange and encourage the development of best practices for deploying solar technology in similar contexts globally. By combining practical implementation with ongoing research and data analysis, the project can continuously evolve and adapt, ensuring its success and sustainability in the long term. The case in Northwest Syria models this, as local health actors have been at the core of humanitarian operations in Northwest Syria, providing contextual and operational knowledge of how to approach remote populations (84). UOSSM currently has partnerships connecting its Syrian staff with researchers at Harvard University, creating databases for the future of health research in Syria (94). Such partnerships are vital, and should be applied to impoverished areas to work toward independence for research systems in conflict zones.

## Discussion

This paper presents the experience of the innovative approach of Syrian humanitarian actors in addressing energy insecurity through renewable solar energy solutions, exemplified by the deployment of a solar-powered electric car to enhance health care delivery and accessibility. This is the first study to describe an implementation of solar-powered electric health vehicles in Syria and offers necessary insights onto the potential successes of such an initiative. This type of humanitarian innovation represents an important step in how humanitarians operate within conflict zones, and renewable avenues for the future to lessen the reliance on natural gas, often a driver for conflict itself. An analysis of UOSSM’s intervention provides critical insights into establishing resilient energy networks that ensure sustainable health care delivery in conflict zones and can derive lessons for future global response.

Globally, the transition to renewable energy has been a necessary point of discussion, especially as it pertains to health systems. Our case study of Syria shows the advantages of a renewable system on reducing operational healthcare costs while also maintaining adequate performance. Having a sustainable source of energy allows healthcare to expand to rural and remote settings, which have otherwise inconsistent power supply. The broader implications of this are mitigation of devastating impacts on healthcare infrastructure due to attacks. Just as a decentralized power grid allows for resilience to attacks on energy, sustainably powering healthcare to reach vulnerable populations allows for a wider scope of healthcare to be practiced: longer operating hours due to availability of lighting and power for medical equipment and increased medical workforce to remote locations since there can be reliable transportation. Similarly, the ability to power refrigerators sustainably will allow for vaccine, blood, and other perishable items to be transported to healthcare settings (95). Increased transport allows for better provision of emergency services, which can allow some healthcare clinics to operate in a secondary or tertiary care capacity- offering mental health, physical rehabilitation, or specialty care.

A common critique of humanitarian innovations in conflict zones is that they are biased externally, often with solutions and decisions coming from the Global North and not consulting with stakeholders in the area of implementation (96, 97). For on the ground solutions to be truly effective, there must be locally-identified solutions, alongside a strategic collaboration with national actors to promote sustainability in the response. In the case of UOSSM’s electric car, a team of doctors and engineers within Syria contributed to its development, and the previously installed solar panels in the Bab El-Hawa hospital in Northern Syria were used to charge the electric vehicles. Community participation in this case is necessary, as it provides a lasting structure to the intervention, as well as long-term cooperation, since Syrians will be the ones utilizing the services. This approach follows numerous recommendations to localize implementation of humanitarian projects (38, 96).

Renewable energy solutions in conflict zones should balance technical innovation with a nuanced understanding of local contexts. Incorporating local expertise in the design and implementation phases ensures the technical feasibility of interventions and addresses cultural and operational challenges (13, 14). For example, training programs for local technicians to manage and maintain solar systems can mitigate the risks of dependency on external actors, while community education initiatives can build trust and acceptance of these systems (98). Scaling such interventions requires a combination of localized solutions, cross-sectoral partnerships, and robust monitoring frameworks to ensure long-term sustainability.

### Conceptual and methodological challenges

There are challenges associated with the existing partnerships that are useful to identify as well. Firstly, the most significant need for conflict to conclude to develop longitudinal systems. A cessation of the violence can change the focus to reconstruction and development, as healthcare workers and civilians would be free to return to their homes (79, 99). In addition to the cessation of violence, there are typically sanctions that are placed on conflict-ridden countries, that can hinder their development and their economies. In Syria, the UN estimated the damage to the economy as a result of the conflict to be at 388 billion dollars, and with the added sanctions by the European Union and the United States on the Syrian economy, it would be difficult to imagine a timely post-war rebuilding phase (47). The other effect of sanctions is the withdrawal of international energy companies as a result of sanctions, due to higher prices and reduced supply of fuel (100). Likewise, this makes it difficult to import solar technology, as well as establish international and inter-agency partnerships, making it difficult to establish a renewable energy system with few existing resources. This has a downstream effect of breeding distrust among citizens of the country, given the isolation from the Global North (101). Without established trust and resource-sharing, this will undermine a country’s ability to create resilient and renewable programming. NGOs, and regional offices should thus create spaces that include stakeholders from regions that are affected by sanctions, whether by establishing committees, creating task groups, or establishing country-specific partners to have established connections to a given region (102).

Logistically, sanctions can also hinder the maintenance of existing renewable energy developments, and conflict can pose a significant security risk when sustaining these initiatives. For example, solar energy is available only during the day, and dependent on the weather conditions and thus geographical location (103). Thus, in order to ensure the upkeep of solar energy cells, it is necessary to have storage devices, which can be expensive even without sanctions in play. To address these challenges, a multi-pronged approach is essential. Leveraging local supply chains and manufacturing can reduce reliance on imports, with local businesses assembling components like batteries or solar panels (104, 105). Where local capacity is limited, regional hubs in stable neighboring countries could serve as assembly and distribution centers. Partnerships with local organizations and NGOs can help navigate security risks and administrative barriers, supporting vehicle deployment, maintenance, and technician training. Advocacy for import exemptions for humanitarian equipment can further streamline operations, ensuring timely deployment in conflict zones. For mitigating security risks, such as solar panel theft, government action is an effective a way to ensure a public service remains active, while maintaining collaboration with local officials (40).

Thirdly, a challenge to implementation of resilient energy systems is the movement of people in conflict and post-conflict settings- among them the many healthcare workers who flee violence. These refugees and IDPs represent a significant human resource that necessarily should have a say in the reconstruction of their countries’ systems. A challenge with implementing solar energy in Syria and elsewhere is the education of households and hospital systems on its maintenance, to ensure long-term usage. Household users of solar PV systems expressed anxiety in the operations and upkeep of their PV systems, while also assuming that their electricity costs would be zero following installation (99). Furthermore, communities may resist transitions to renewable energy, seeing it as negatively affecting their livelihoods, as was the case in Morocco for example. The Moroccan Agency for Sustainable Energy (MASEN) attempted to build their solar project on common land belong to a herder community, who initially resisted due to it affecting the lands they use for their livestock (106, 107). Addressing these challenges requires not only technical training and community education but also inclusive decision-making processes that engage displaced populations and local communities to foster acceptance and long-term sustainability. This intervention provides a template for integrating renewable energy into health care systems in other conflict-affected regions, demonstrating how local and global collaboration can address similar challenges.

## Conclusion

The development of a solar-charged electric car in Syria exemplifies how renewable energy solutions can transform health service delivery in conflict zones, providing a model that can be adapted and scaled globally to enhance health system resilience. The prolonged conflict lasting for over 13 years has resulted in unprecedented devastation, displacement, and loss of life. In this context, the strategic utilization of an abundant renewable resource plays a critical role in reducing reliance on natural gas and offering a promising pathway toward addressing critical health needs in a humanitarian setting. In prioritizing a sustainable solution to address the challenges of a fractured health system, UOSSM’s SSI demonstrates the impact a collaboration between humanitarians and local communities can have while prioritizing sustainability and autonomy for the Syrian population, and these lessons can extend further to other conflict areas globally. The benefits of renewable energy have been extensively documented, and it is important to develop the ability to independently maintain and scale renewable energy systems, ensuring sustainability and resilience in health care delivery across conflict-affected regions. To facilitate broader adoption of renewable energy solutions in conflict zones, policymakers and humanitarian agencies should integrate solar energy infrastructure into emergency response frameworks. This requires fostering cross-sectoral collaborations, ensuring regulatory flexibility for importing renewable technology, and investing in long-term capacity-building programs for local technicians and health facilities. Additionally, further research in contexts beyond Syria would be valuable to advance our understanding of renewable energy innovations tailored to improve health care transport in active conflict settings facing similar challenges.

## Data Availability

The raw data supporting the conclusions of this article will be made available by the authors, without undue reservation.
